# The Impact of Single-Level ACDF on Neural Foramen and Disc Height of Surgical and Adjacent Cervical Segments: A Case-Series Radiological Analysis

**DOI:** 10.3390/brainsci13010101

**Published:** 2023-01-04

**Authors:** Rosario Maugeri, Lara Brunasso, Andrea Sciortino, Alba Scerrati, Felice Buscemi, Luigi Basile, Giuseppe Roberto Giammalva, Roberta Costanzo, Francesco Bencivinni, Eleonora Bruno, Domenica Matranga, Laura Maniscalco, Francesco Gioia, Silvana Tumbiolo, Domenico Gerardo Iacopino

**Affiliations:** 1 Neurosurgical Clinic, Post Graduate Residency Program in Neurologic Surgery, Department of Biomedicine Neuroscience and Advanced Diagnostics, School of Medicine, University of Palermo, 90133 Palermo, Italy; 2 Department of Neurosurgery, Sant’Anna University Hospital of Ferrara, 44124 Ferrara, Italy; 3 Department of Biomedicine, Neurosciences and Advanced Diagnostic, Unit of Radiology, University of Palermo, 90133 Palermo, Italy; 4 Health Promotion, Mother and Child Care, Internal Medicine and Medical Specialties, University of Palermo, 90133 Palermo, Italy; 5 Department of Radiology, Villa Sofia—Cervello Hospital, 90146 Palermo, Italy; 6 Department of Neurosurgery, Villa Sofia—Cervello Hospital, 90146 Palermo, Italy

**Keywords:** ACDF, cervical foramen, foramen height, cervical spondylosis, foraminal stenosis, cervical cage, intervertebral distraction, neural decompression

## Abstract

Background: ACDF has become one of the established procedures for the surgical treatment of symptomatic cervical spondylosis, showing excellent clinical results and effective improvements in neural functions and neck pain relief. The main purpose of ACDF is neural decompression, and it is considered by some authors as an indirect result of the intervertebral distraction and cage insertion and the consequent restoration of the disc space and foramen height. Methods: Radiological data from 28 patients who underwent single-level ACDF were retrospectively collected and evaluated. For neural foramen evaluation, antero-posterior (A-P) and cranio-caudal (C-C) diameters were manually calculated; for intervertebral disc height the anterior, centrum and posterior measurement were calculated. All measurements were performed at surgical and adjacent (above and below) segments. NRS, NDI and also the mJOA and Nurick scale were collected for clinical examination and complete evaluation of patients’ postoperative outcome. Results: The intervertebral disc height in all its measurements, in addition to the height (C-C diameter) of the foramen (both right and left) increase at the surgical segment when comparing pre and postop results (*p* < 0.001, and *p* = 0.033 and *p* = 0.001). NRS and NDI radiculopathy scores showed improved results from pre- to post-op evaluation (*p* < 0.001), and a negative statistical correlation with the improved disc height at the surgical level. Conclusions: The restoration of posterior disc height through cage insertion appears to be effective in increasing foraminal height in patients with symptomatic preoperative cervical foraminal stenosis.

## 1. Introduction

Cervical radiculopathy is a common clinical scenario, and a worldwide cause of neck pain, causing different grades of disability and a consequent impact on both the working life and on quality of life in general. Patients usually complain of a broad range of clinical manifestations, and they may include neck pain associated with radiation into the ipsilateral arm in the affected nerve-root distribution, and a combination of paresthesia, sensory deficits, motor deficits, and diminished reflexes [1,2]. Although many different theories have been proposed as to its cause, including intraneural edema and demyelination due to obstruct blood flow, on a morphological basis cervical radiculopathy derives from a mechanical compression of the cervical nerve root. The compression may be due to a combination of factors including disc herniation or bony osteophytes that impinge on the cervical nerve root, and the consequent nerve damage derives from both mechanical (localized ischemia) and chemical (proinflammatory cascade with sensitization and increased pain) pathways [2,3,4,5]. The majority of cervical radiculopathies result from degenerative cervical spondylosis rather than soft disc herniation [6]. Cervical spondylosis refers to the age-related degenerative changes that occur in the cervical spine, and where the commonly called wear and tear of the cervical spine leads to decreased disc height and subsequent bony hypertrophy resulting from increased loads through the Luschka intervertebral joints anteriorly and zygapophyseal joints posteriorly, in addition to foraminal narrowing [2]. When conservative treatment fails in relieving pain or when numbness and/or weakness develop, a surgical approach aims to decompress the affected nerve root. Smith and Robinson first introduced the technique [7], and currently, with a lot of technological improvements (such as effective intervertebral distraction with Caspar plating system), ACDF has become one of the established procedures for the surgical treatment of cervical spondylosis [8,9,10,11]. Literature reports outline that ACDF produces excellent clinical results with effective improvements in neural functions and neck pain relief detrimental to health-related quality of life; but controversy exists about the role of the implanted intervertebral cage in increasing the area and/or height of the surgical segment foramen. As the main purpose of ACDF is neural decompression, many authors agree on considering the clinical benefit as an indirect result of the intervertebral distraction and cage insertion, and the restoration of the disc space and foramen height. In fact, an increase in the foraminal area from pre to postoperative measurements was widely reported [12,13,14,15,16]. This is contrary to the findings other authors, who believe that there is no evidence to indicate that an increase in the disc height with ACDF technique affects increased the long-term neural foramen area of the surgical segment, and that symptoms may resolve due to the removal of soft disc, extraforaminal bony decompression or stabilization of the motion segment [17]. Moreover, there remains a lack of information about the behavior and repercussions for the foramen of the adjacent motion segments, and how the variation in disc height may influence them and the long-term clinical response.

In a double-center case series of patients who underwent ACDF for cervical radiculopathy, the present study investigated the radiological impact of the implant of a single-level cage on the foramen dimensions of the surgical segment. Moreover, the authors aimed to assess the impact of the cage implantation on the foramen of the adjacent segments, including how the foramen above and below the surgical segment varies as the disc height changes, and the impact of the restored disc height of the surgical segment on the disc height of the adjacent (both above and below) segments by three measurement parameters (anterior, intermediate and posterior height of the disc). Patient-reported questionnaires were applied to estimate clinical outcomes concerning treatment efficacy and functional disability following ACDF.

## 2. Materials and Methods

### 2.1. Patient Sample and Patient Selection

The present retrospective study included 28 patients who underwent single-level ACDF for cervical radiculopathy in the neurosurgical department of two different Italian hospitals from January 2018 to April 2022. The inclusion criteria were the following: (1) cervical single-level involvement confirmed by preoperative imaging (MRI and CT) and physical examination; (2) single-segment ACDF surgery; (3) cage implantation; and (4) available pre- and post-operative cervical spine CT scans. According to strict exclusion criteria, patients were excluded from the present retrospective analysis if: (1) two or more segments were involved; (2) pre- and/or post-cervical CT scans were not available, (3) previous cervical surgeries (both anterior or posterior approach); (4) concomitant cervical spine fractures or dislocation; and (5) tumor compression presence, diagnosed infections, or multilevel OPLL. Medical history, neurological and clinical examination, and radiological studies (cervical CT scans) were collected for each patient in order to perform the statistical analysis.

### 2.2. Surgical Approach

All patients underwent single-level ACDF surgery performed by three senior neurosurgeons in two different hospital centers. The patients were placed in supine decubitus with head fixed in mild hyperextension. After level check supported by preoperative fluoroscopy, a right anterolateral cervical longitudinal skin incision was performed. A blunt dissection of the subcutaneous tissues and muscle layers was performed with the usual technique. The anatomic corridor bordered medially by the esophagus and trachea, and laterally by the sternocleidomastoid muscle and the neck vascular-nervous bundle was followed until the deep cervical fascia and the pre-vertebral layer was reached. After an intraoperative fluoroscopic check of the surgical level, the annulotomy was performed. Caspar’s screws were placed on the soma of the vertebra one level above and one below the surgical disc level, and the surgical intervertebral space was distracted and opened. Microdiscectomy was accomplished using rongeurs and curettes of different sizes and angles, until visualization of the posterior longitudinal ligament with its removal in order to facilitate expansion of the disk height. The choice of cage size depended mainly on the height of the adjacent less degenerative intervertebral disc on the sagittal view. All surgeons implanted the same type of cage. After the evaluation of the optimal size of a trial cage, the placement of an appropriated-sized cage was performed containing synthetic bone to facilitate interbody arthrodesis. Proper placement was verified by intraoperative fluoroscopic control, and the Caspar distractor was released. The layered suturing of soft tissues was accomplished after the placement of an 8-mm submuscular drainage. A post-operative cervical CT was performed on the day following surgery. All patients wore a rigid cervical collar for 4–6 weeks post treatment.

### 2.3. Radiological Parameters and Analysis

Cervical CT scan collections and radiological measurements were performed in cooperation with the Department of Radiology of both hospitals. All measurements were taken by the same author (A.S.), and, in a second step, checked by each of the three radiologists among the authors (F.B., E.B., F.G.), separately. CT post-processing procedures, such as MPR (multiplanar reconstruction) reconstruction and MIP (maximum intensity projection) images, were applied to the collected cervical CT scans in order to minimize errors and consequential starting bias. The aim of the study is to investigate the effect of cage implantation on surgical levels and adjacent segments, and assess how foramina and intervertebral space on these levels may be influenced and how this phenomenon may affect patients’ clinical conditions.

**Figure 1:**
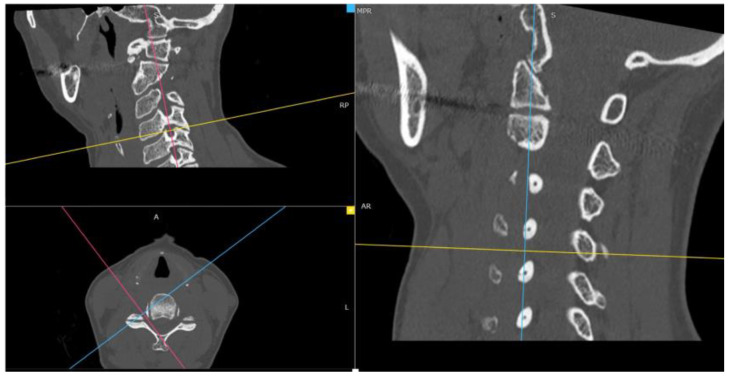
Neural cervical foramen was centered using MIP and MPR CT reconstructions.

**Figure 2:**
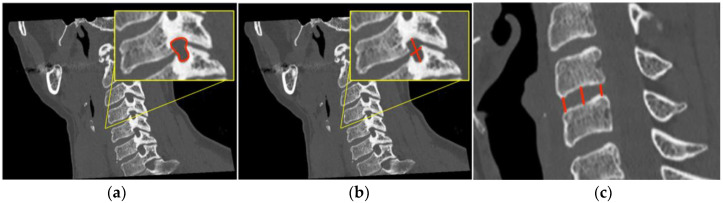
In (**a**) the outline of the foramen is drawn in yellow and in (**b**) the measurement of the intervertebral foramen antero-posterior (A-P) and cranio-caudal (C-C) diameters are drawn in red, which means foramen width and height, respectively, are shown with red lines. In (**c**) the red lines show the antero, centrum and postero disc height measurement.

Through the use of MIP and MPR cervical bone-window CT scans, the correct site for measurements at each cervical segment (surgical or above or below) was selected matching the midline point on the anterior surface of the vertebral body on coronal view with the intervertebral foramen plane on axial view, and then adjusted with a plane parallel to the inferior endplate of the level above on the sagittal view. (Figure 1). The antero-posterior (A-P) and cranio-caudal (C-C) diameters of each foramen (surgical and adjacent levels, right and left) were manually measured (Figure 2a). The measurement of the C-C diameter was considered similar to the height of the foramen, and in sagittal reconstruction it was the distance between the midpoint of the upper and lower corresponding pedicles; the measurement of the A-P diameter was considered similar to the width of the foramen, and in sagittal reconstruction it was the distance between the anterior and the posterior border of the inferior intervertebral notch. For the intervertebral disc measurement, the disc height was measured in the anterior, middle, and posterior third on sagittal CT scans. The measure of the intervertebral disc space was considered from the corresponding point (anterior, middle, and posterior) of the inferior endplate of the upper vertebral body to the superior endplate of the lower vertebral body (Figure 2b). CT scans were performed before and within 72 h after surgery, usually the day after the procedure.

For each patient the following parameters have been calculated: (1) the measurement of preoperative and postoperative A-P diameter and C-C diameter of the intervertebral foramen bilaterally at each level (affected level, the one below and the one above); (2) measurement of preoperative and postoperative height (anterior, middle and posterior) of the intervertebral disc at each level (affected level, the one below and the one above).

### 2.4. Clinical Evaluation

A thorough preoperative and postoperative neurological examination was performed for each patient in order to estimate recovery and any improvement in the neurological status. The analysis of the clinical condition was based on the administration of validated tests to assess patients’ quality of life and clinical condition. NRS (Numeric pain Rating Scale) and NDI (Neck Disability Index) defined scales for radiculopathy were evaluated in the pre- and post-operative period. mJOA (modified Japanese Orthopedic Association) and Nurick’s were also examined for a comprehensive evaluation of the patients’ clinical and neurological status.

### 2.5. Statistical Analysis

Continuous variables were summarized through mean, standard deviation (SD) and with median and interquartile range (IQR). A one-way ANOVA test with repeated measures, followed by planned comparisons, was applied to evaluate the paired preoperative and postoperative differences as related to the location of the cage (the operated vertebra, and in the above and below adjacent segments), to the height of the disk space (anterior, medium and posterior) and to the diameter of the foramen (left and right). Statistical analysis was performed using R software (version 4.0.2) and an alpha value of 0.05 was considered significant.

## 3. Results

Twenty-eight patients were evaluated including 11 females and 17 males (M/F ratio of 1.54) with a mean age of 58.46 years old (at the time of evaluation) and a standard deviation (SD) of 11.24. The surgical segment was C3–C4 in three patients (10.7%), C4–C5 in ten patients (35.7%), C5–C6 in five patients (17.8%), and C6–C7 in ten patients (35.7%). (Table 1)

As shown in Table 2, the measurement of preoperative and postoperative height of the intervertebral disc is statistically significant only for the operated disc (*p*-value < 0.001).

Regarding the diameter, statistically significant differences were found for the right and left cranio-caudal diameter of the central foramen (right mean difference = 0.506, *p*-value = 0.033, left mean difference = 0.810, *p*-value = 0.001) and for the anterior-posterior diameter of the left foramen of the segment above (mean difference= 0.406, *p*-value = 0.020) (Table 3).

**Figure 3:**
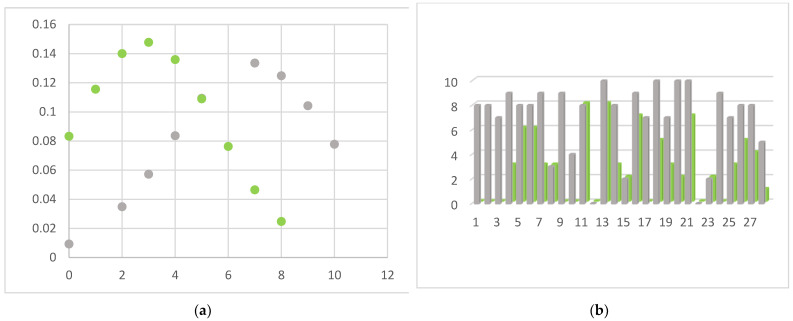
Pre- (grey) and post-operative (green) graphic representation of NRS evaluation in our cohort. In (**a**) the center of the peak between pre- and post-operative value there is a shift from 7 (preoperatively) to 3 (postoperatively). Description of what is contained in the first panel; in (**b**) it is shown how in most cases NRS scores were reduced after surgical treatment, meaning a reduction in pain levels.

**Figure 4:**
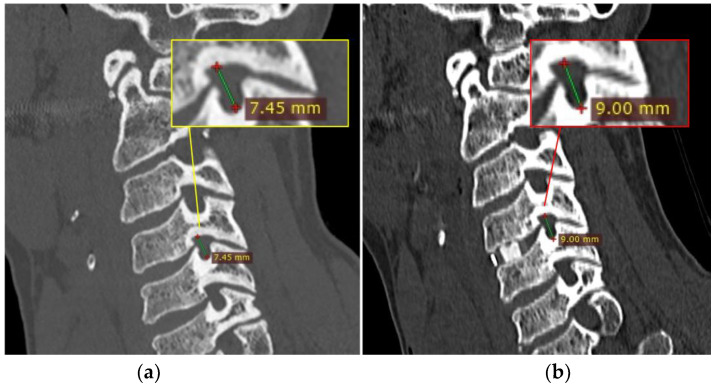
Case illustration. Pre-(**a**, yellow box) and post-(**b**, red box) operative comparison of the foramen height (C-C diameter) at C4-C5, on the right side for illustrative purposes only, after the implant of a single-level cage.

Presenting symptoms included neck pain, radiculopathy and signs of cord involvement without gait impairing 14 patients (Nurick grade 0, I and II) and mild/moderate/severe myelopathic symptoms in 14 patients (Nurick grade III, IV, V or VI). According to mJOA score, no myelopathy was found in two patients, mild myelopathy (mJOA score from 15 to 16) in 10, moderate myelopathy (mJOA score from 12 to 14) in eight, and severe myelopathy (mJOA score from 0 to 11) in 8. An NRS and NDI evaluation between the pre- and post-operative period showed statistically significant results (*p* value < 0.001). (Figure 3) A negative correlation was confirmed between postoperative clinical outcome and postoperative disc height in its centrum measurement, and specifically −0.468 (*p*-value = 0.011) with the NRS scale and −0.603 (*p*-value < 0.001) with the NDI score. Complications of the ACDF procedure are not in the scope of this article, but we found that 60% of patients suffered mild temporary dysphagia and 15% suffered from slight temporary dysphonia. No postoperative hematomas or cage displacement were documented in our series. No permanent deficits were recorded, and temporary disturbances progressively regressed before discharge. An illustrative case is shown in Figure 4, comparing pre- and post-operative results. Table 4 shows patients’ demographic data, disease level, clinical pre- and post-operative grade, and a brief description of onset symptoms.

## 4. Discussion

When cervical radiculopathy is present with recognized nerve root encasement/compression on diagnostic imaging and conservative therapeutic measures have failed, ACDF demonstrated high-evidence results to improve neurological symptoms with rapid relief and long-term maintenance, and this is considered the gold standard for its surgical treatment [11,18]. As argued, surgical treatment for cervical spondylosis and foraminal stenosis in symptomatic cervical radiculopathy aims to decompress the affected nerve root(s). The grade of recognizable cervical foraminal stenosis has not been clearly defined in the literature. The foraminal width has been considered an important factor for evaluating the intervertebral foramen stenosis status before surgery, therefore oblique X-ray and/or multiparametric CT scans are widely used to identify and assume whether the canal of the nerve root is narrow or not; nevertheless, the results and measurements are still not consistent and defined. Some authors, for example, experienced that even patients with excessive formation of uncinate process osteophytes and radiologically evident foraminal stenosis might not present with any clinical symptoms [19]. The cervical bony foraminal width and height has been investigated in anatomical studies, and it was documented that nerve roots take up an average of 2–35% of the available area of the intervertebral foramen in the neutral position [20,21], so that, despite the existence of factors reducing effective foraminal space, there is still adequate space for the nerve root [22]. Interesting correlations between the foraminal opening and flexion/extension movements were evaluated [17] and are worthy of further attention but are not in the scope of the present article. 

Sun and colleagues [19] accomplished one of the first studies to evaluate the potential relationship between preoperative width of the cervical intervertebral foramen and patients’ clinical outcome. A previous study found that the average bony foraminal width and height were 6–7 mm and 8–11 mm, respectively [20]. The authors [19] demonstrated with their analysis that persistent pain would be more likely to occur if the value of the foraminal width was equal to or less than 4.35 mm; in contrast, the possibility of persistent pain dramatically decreased when the preoperative foraminal width was over 4.35 mm. In performing ACDF, the distraction of disc space is an integral and fundamental part of the procedure, which could contribute to optimize and visualize decompression, to favor the insertion and positioning of the cage, and lastlyto recover the disc height. An indirect benefit on foraminal height restoration from increased intervertebral disc space is widely reported in the literature [12,13,23,24]. However, it is also well-documented that excessive distraction may lead to increased mechanical stress between the upper and lower segments, and for example consequent adjacent segment degeneration (ASD) and pathological endplate changes could be some of the major long-term failures of ACDF surgery [25]. Accordingly, the intervertebral distraction also has an impact on the intervertebral neural foramen, and previous studies showed that when the height of the bone graft increases to a certain point, the size of the surgical foramen would begin to decrease [23]. The study of adjacent segmental foramen has received insufficient attention. The working group of Wu [24] found that significant improvement with the height and area of the intervertebral foramen in the surgical segment was approved after the operation and at the last follow-up with a *p* < 0.05, and that the area and height of the surgical segment foramen and the degree of intervertebral distraction were positively correlated. Moreover, their data demonstrated that the foraminal area and height at the adjacent levels were reduced in one group of patients where the intervertebral distraction degree was >1.40, and these measurements and the degree of intervertebral distraction were negatively correlated. The authors argued that their results, consistent with the examined literature, might be the result that an oversized cage would contribute to over the distraction of intervertebral space. Yang et al. [23] found that with the increase of the intervertebral space distraction, the size of the intervertebral foramen began to decrease until a certain point, concluding that 160% of the mean height of the adjacent intervertebral spaces was the optimal degree of distraction. Wu et al. [24] concluded that 1.20~1.80 fold distraction was the optimal range of intervertebral distraction after cage insertion. Table 5 shows the results of articles about the radiological and clinical (when known) impact of the implant of a single-level cage on the foramen dimensions of the surgical segment in the most updated literature; only one study also evaluated the impact on the foramen dimensions of the adjacent segments.

The purpose of the present study was to evaluate the effect on surgical and adjacent segment foramen diameters and disc height after implanting a single-level cervical cage. The results presented are preliminary reports and part of an ongoing thorough radiological multicentric assessment of a larger sample. All patients underwent the ACDF approach because cervical radiculopathy was correlated with foraminal stenosis, and the stenosis was correlated with other degenerative cervical spine components including the reduction of the intervertebral disc space; therefore, cages were used to restore this disc space height, consistent with surgical indications and literature results already mentioned. Compared with the outdated insertion of a bone graft, surgical insertion of a cage with all advanced technical support has widely been demonstrated to be advantageous for avoiding the risks of the early collapse of graft bones before fusion with recurrence of neurological symptoms, and for being adaptable with a wide variety of sizes and the surgeons’ choice. Additional anterior plating was unnecessary because vertebral instability signs were not documented on preoperative imaging. Pre- and post-operative CT scans were performed in all patients to take measurements before and after cage implantation in ACDF. Previous studies used only mid-sagittal reconstruction images to perform all measurements; we underline the importance of using CT post-processing procedures, such as MIP and MPR, in order to avoid simple bias during image acquisition. In the current study, surgical treatment with cervical cage implantation for foraminal stenosis led to statistically significant results in restoring disc height at the surgical segment (*p* < 0.001), and in enlarging the C-C diameter of the right and left foramen of the surgical segment (*p* = 0.033 and *p* = 0.001, respectively), which means an increase in the foramen height at the surgical level. The height of the surgical segment foramen and the degree of intervertebral distraction were positively correlated. A statistically significant value was also found also for the A-P diameter (width) of the left foramen of the segment above, but the meaning of which is actually clinically or pathophysiological unclear, and it might become the object of future studies with higher numbers and comparisons. The evaluation of the above-defined objective scale and score, such as postoperative neck pain and arm pain through NRS and NDI score, showed improvement (*p* value < 0.001), and a negative correlation between postoperative neck and arm pain and disability and the postoperative disc height was also documented, which indicates the greater relief of neck and arm pain together with better postoperative recovery with a greater increase in the disc height after cage implantation.

### Limitations

Study limitations need to be considered when interpreting the results of the present study. First, this is a retrospective study and it is subject to all the inherent biases in this study design. Thus, it is unable to account for some confounding factors such as various procedures of physical therapy, psychosocial determinants, and other variables that may influence the collected clinical score. Furthermore, some postoperative factors, such as the application of drugs, working pressure, and other potential factors that may affect self-rated disability scores, were not evaluated. Second, the small sample size and manual measurements may lead to misinterpretations of the present results. Third, all procedures were performed by three surgeons at two individual institutions, which may limit the generalizability of the results. Fourth, no longer follow-up period was considered, which may provide better evidence that explains the impact of foraminal dimensional changes over time. Fifth, in line with what was mentioned in the discussion, all CT images were acquired in a relaxed supine position, which eliminated the effects due to dynamic morphological changes in the flexion and extension of the foraminal dimensions.

## 5. Conclusions

In the present work, we found that single-level cervical cage implantation has a positive correlation with the increase in the disc height and the increase in the foraminal height at the surgical level, evaluated by the antero, centrum and postero disc measurement and by the C-C diameter, respectively. Thus, in our experience the restoration of posterior disc height appears to be necessary to enlarge the foraminal dimension in patients with symptomatic preoperative cervical foraminal stenosis. No significant results were found examining pre- and post-operative findings for surgical adjacent segments (above and below). Our results appear to be consistent with some of the literature, and ongoing retrospective multicentric studies with greater numbers will demonstrate more a extensive analysis.

## Figures and Tables

**Table 1:** Surgical level rate.

Surgical Level	%
C3-C4	10.7
C4-C5	35.7
C5-C6	17.8
C6-C7	35.7

**Table 2:** Statistical results from radiological analysis between pre and postoperative disc height.

	PRE		POST			
	Mean (SD)	Median (IQR)	Mean (SD)	Median (IQR)	Mean of Differences	*p*-Value
Above_antero	4.50 (1.22)	4.40 (3.60–5.35)	4.75 (0.99)	4.60 (4.00–5.55)	0.248	0.243
Above_centrum	5.37 (1.10)	5.36 (4.60–6.10)	5.36 (1.05)	5.25 (4.83–5.95)	−0.004	0.948
Above_postero	3.74 (0.96)	3.70 (3.20–4.43)	3.99 (0.97)	4.15 (3.25–4.64)	0.250	0.328
Surgical_antero	4.14 (1.81)	4.15 (2.48–4.90)	8.04 (2.01)	7.95 (7.00–9.30)	3.893	<0.001
Surgical_centrum	4.54 (1.32)	4.25 (3.48–5.32)	7.30 (1.27)	7.20 (6.30–7.93)	2.763	<0.001
Surgical-postero	3.53 (1.06)	3.50 (2.95–4.50)	6.79 (1.87)	6.65 (5.93–8.15)	3.261	<0.001
Below_antero	4.53 (1.73)	4.40 (3.38–5.90)	4.65 (1.61)	4.59 (3.38–5.60)	0.120	0.551
Below_centrum	5.02 (1.38)	5.35 (3.92–6.03)	5.23 (1.36)	5.25 (4.27–6.43)	0.212	0.142
Below_postero	3.38 (1.01)	3.60 (2.65–4.00)	3.52 (1.04)	3.60 (2.72–4.23)	0.143	0.315

**Table 3:** Statistical results from radiological analysis between pre and postoperative foraminal diameters of the surgical and adjacent (above and below) segments.

	PRE		POST			
	Mean (SD)	Median (IQR)	Mean (SD)	Median (IQR)	Mean of Differences	*p*-Value
A-P right_above	5.62 (1.47)	5.65 (4.90–6.38)	5.73 (1.39)	5.35 (4.90–6.6)	0.108	0.532
A-P left_above	5.43 (1.51)	5.60 (4.60–6.46)	5.84 (1.42)	5.95 (4.76–6.76)	0.406	0.020
C-C right_above	9.33 (1.66)	9.30 (7.97–10.67)	9.75 (1.44)	9.60 (8.95–10.62)	0.425	0.059
C-C left_above	9.59 (1.29)	9.25 (8.70–10.65)	9.75 (1.51)	9.85 (8.52–10.55)	0.160	0.484
A-P right_surgical	5.29 (1.48)	5.30 (4.07–6.35)	5.15 (1.35)	5.05 (4.30–5.67)	−0.145	0.401
A-P left_surgical	5.15 (1.31)	5.20 (4.32–5.85)	5.23 (1.18)	5.32 (4.55–6.32)	0.073	0.659
C-C right_surgical	8.86 (1.45)	8.62 (8.10–9.10)	9.36 (1.77)	9.45 (8.10–10.38)	0.506	0.033
C-C left_surgical	8.62 (0.96)	8.45 (7.88–9.25)	9.43 (1.53)	9.15 (8.28–10.80)	0.810	0.001
A-P right_below	5.89 (1.46)	5.60 (4.65–7.03)	5.59 (1.73)	5.55 (4.20–7.12)	−0.298	0.063
A-P left_below	5.86 (1.46)	5.95 (4.97–6.43)	6.13 (1.54)	6.00 (5.05–7.30)	0.271	0.075
C-C right_below	9.36 (1.46)	9.20 (8.45–10.10)	9.45 (1.46)	9.38 (8.17–10.50)	0.090	0.717
C-C left_below	9.18 (1.36)	9.45 (6.00–11.30)	9.48 (1.43)	9.35 (8.67–10.70)	0.300	0.173

**Table 4:** Patients’ demographic data and clinical evaluation of the present case series.

Age, Sex	Surgical Level	mJOA (Preop; Postop)	NDI (Preop; Postop)	NRS (Preop; Postop)	Cage Size	Onset Symptoms
67, F	C5-C6	14; 16	34; 18	8; 9	6 mm	Bilateral cervicobrachialgia and paresthesia
48, F	C4-C5	16; 18	30; 1	8; 0	5 mm	Intense cervicalgia and left brachialgia
48, F	C4-C5	14; 18	36; 4	7; 0	5 mm	Cervicalgia, left upper limb hyposthesia, hyperreflexia
63, M	C4-C5	15; 17	27; 10	9; 3	5 mm	Cervicalgia and left brachialgia
76, F	C4-C5	10; 12	38; 31	8; 6	6 mm	Spastic paraparesis, hyperreflexia, upper limbs motor weakness
71, F	C4-C5	8; 16	43; 25	8; 6	5 mm	Spastic paraparesis, hyperreflexia, upper limbs motor weakness
42, M	C6-C7	14; 17	18; 3	9; 3	6 mm	Intense cervicalgia and right brachialgia
64, M	C6-C7	16; 18	12; 2	3; 3	5 mm	Left upper limb motor weakness
47, M	C5-C6	13; 18	35; 3	9; 0	5 mm	Intense cervicalgia, right brachialgia and hypoesthesia
65, M	C6-C7	17; 18	13; 0	4; 0	5 mm	Left upper limb mild hypoesthesia
46, F	C5-C6	15; 16	21; 18	8; 8	5 mm	Cervicalgia, walking disturbances, hyperreflexia, bilateral upper limb motor weakness
75, M	C3-C4	9; 15	36; 28	0; 0	5 mm	Hyperreflexia, bilateral upper limb motor weakness, walking disturbances
52, M	C3-C4	10; 13	41; 34	10; 8	6 mm	Intense cervicalgia, right brachialgia
41, M	C6-C7	17; 18	15; 7	8; 3	5 mm	Right upper limb mild hypoesthesia
63, M	C6-C7	13; 14	31; 26	2; 2	5 mm	Spastic paraparesis, bilateral prehensile motor deficit
45, F	C6-C7	15; 17	26; 12	9; 7	5 mm	Right upper limb brachialgia and mild hypoesthesia
44, M	C4-C5	16; 17	29; 15	7; 0	6 mm	Intense cervicalgia, right brachialgia and hypoesthesia, hyperreflexia
57, M	C6-C7	6; 11	43; 11	10; 5	5 mm	Intense bilateral brachialgia, mild walking disturbances
73, M	C4-C5	16; 17	23; 7	7; 3	5 mm	Right upper limb brachialgia and mild hypoesthesia
49, F	C5-C6	14; 15	33; 8	10; 7	5 mm	
52, F	C6-C7	16; 18	35; 5	10; 2	6 mm	Intense cervicalgia, left prehensile motor deficit
59, F	C5-C6	15; 15	4; 0	0; 0	5 mm	Bilateral prehensile motor deficit, diffuse upper limbs hypoesthesia
70, M	C6-C7	10; 12	38; 33	2; 2	5 mm	Mild spastic paraparesis, urinary incontinence
67, M	C6-C7	15; 17	37; 5	9; 0	5 mm	Intense cervicalgia, left prehensile motor deficit
70, M	C4-C5	11; 13	43; 38	7; 3	5 mm	Spastic paraparesis, bilateral prehensile motor deficit, urinary incontinence
73, M	C4-C5	12; 14	20; 7	8; 5	6 mm	Intense bilateral brachialgia, hyperreflexia, urinary incontinence
60, M	C3-C4	1; 2	47; 45	8; 4	6 mm	Cervicalgia, severe spastic paraparesis, hyperreflexia, bilateral clonus, urinary incontinence
50, M	C4-C5	12; 15	29; 10	5; 1	6 mm	Right upper limb brachialgia and mild hypoesthesia

**Table 5:** Summary of the most updated literature search concerning the radiological and clinical impact of a single-level cage on the cervical foramen dimension.

Authors, Year	N° of Patients	Type of Study	Clinical Parameter	Pre and Postop Radiological Parameter	Results
Wu C et al., 2021 [24]	61	Retrospective	VAS, mJOA	Intervertebral disk height of level treated and upper and lower levels (and ratio); Regional curvature and global curvature of cervical spine; Height and area of the neural foraminal of level treated, lower and upper levels.	Positive correlation between pre and postop intervertebral disk ratio and pre and postop area and height of neural foramina of the surgical segment; Improvement of postoperative mJOA and VAS scores.
Sun B et al., 2021 [19]	538	Retrospective	NDI, mJOA, BMI	Width of the intervertebral foramen of treated segment; Height of the intervertebral foramen; Only preoperative C2-C7 Cobb angle.	Pain relief was negatively affected by the symptomduration and ratio of disc space distraction; An increase in the preoperative width of the intervertebral foramen decrease the possibility of persistent pain.
Abudouaini H et al., 2021 [26]	148	Retrospective	VAS, mJOA, NDI	Intervertebral height; Cervical curvature; Functional spine unit (FSU); Intervertebral foramen diameter.	No clear correlation between IH changes and clinical efficacy within a year of surgery; If postoperative IH changes are maintained at 2 to 4 mm after a year, satisfactory imaging parameters and relatively low complications using a zero-profile device
Suk K-S et al., 2015 [27]	44	Prospective	VAS, NDI, donor site pain, subjective improvement rate	Anterior and posterior disk height; Height anterior-posterior diameter of the foramen; Cobb angle.	Foraminal dimension was negativelycorrelated with the arm pain; Restoration of posterior diskheight was necessary to widen the foraminal dimension; Increased lordosis of the fusion segment did not help to widenthe foraminal dimension.
Sekerci Z et al., 2006 [28]	20	Prospective	N/A	Mean height of neural foramina; Mean height of disk space.	Restoring foraminal height and maintenance of stability achieved by using implantation of PEEKcage containing synthetic bone; Use of synthetic graft material allowed for shorthospital stays and avoided donor site-related complications.
Bartels RHM et al., 2001 [29]	13	Prospective	N/A	Mean angle between the two adja-cent endplates; Mean height of neural foramina.	Height of neural foramina increased 1 year after surgery; Angle between two adjacent endplates increased postop (improvement of cervical lordosis).

## Data Availability

Not applicable.
